# Developing indicators of pattern identification in patients with stroke using traditional Korean medicine

**DOI:** 10.1186/1756-0500-5-136

**Published:** 2012-03-13

**Authors:** Ju Ah Lee, Tae-Yong Park, Jungsup Lee, Tae-Woong Moon, Jiae Choi, Byoung-Kab Kang, Mi Mi Ko, Myeong Soo Lee

**Affiliations:** 1 Brain Disease Research Center, Korea Institute of Oriental Medicine, 1672 Yuseongdae-ro, Yuseong-gu, Daejeon 305-811, Republic of Korea; 2 National Rehabilitation Center, Seoul, South Korea

**Keywords:** Pattern identification, Indicator, Stroke, Standardization, TKM

## Abstract

**Background:**

The traditional Korean medical diagnoses employ pattern identification (PI), a diagnostic system that entails the comprehensive analysis of symptoms and signs. The PI needs to be standardized due to its ambiguity. Therefore, this study was performed to establish standard indicators of the PI for stroke through the traditional Korean medical literature, expert consensus and a clinical field test.

**Methods:**

We sorted out stroke patterns with an expert committee organized by the Korean Institute of Oriental Medicine. The expert committee composed a document for a standardized pattern of identification for stroke based on the traditional Korean medical literature, and we evaluated the clinical significance of the document through a field test.

**Results:**

We established five stroke patterns from the traditional Korean medical literature and extracted 117 indicators required for diagnosis. The indicators were evaluated by a field test and verified by the expert committee.

**Conclusions:**

This study sought to develop indicators of PI based on the traditional Korean medical literature. This process contributed to the standardization of traditional Korean medical diagnoses.

## Background

Pattern identification is a system of diagnosis in traditional Korean medicine (TKM) that is characterized by its own theoretical basis and practical experience [1]. This unique system entails a comprehensive symptom analysis and an investigation of the illness, its cause and nature, the patient's physical condition and the patient's treatment through four examinations (inspection, listening and smelling, inquiry and palpation) [2]. TKM has advantages, such as one-to-one personalized care accompanying the patient's diagnosis. However, these characteristics are criticized because of the ambiguous process of diagnosis. Different ways of pattern identifications are often used for diagnosis by different Oriental medical clinicians in identical patients [3]. Oriental medical clinicians have claimed that differences exist between Western medicine and TKM in terms of therapy and the objective for treating and diagnosing patients. However, standardized and objective methods for diagnosis in TKM are needed. The Korea Institute of Oriental Medicine (KIOM) has conducted a fundamental study for the standardization and objectification of pattern identification in TKM for stroke (SOPI-Stroke) since 2005 [4-6]. We organized a committee comprised of physicians and researchers to draft a standardized document for pattern identification in stroke (D-SPI). This research concerned the process of developing standard indicators of pattern identification. This is a primary step of collecting and sorting clinical data toward further investigation on standardization of the pattern identification.

## Methods

### Collecting of pattern indicators

To determine important pattern indicators, the KIOM research team conducted manual searches of 10 academic sources: (1) the Dongeuibogam [7]; (2) A study of the standardization of diagnoses and diagnostic requirements in traditional Korean medicine II [8]; (3) Traditional Korean medicine diagnostics [9]; (4) Pattern identification diagnostics [10]; (5) Traditional Korean medicine pathology [11]; (6) A Standard form of pattern identification for stroke patients [12]; (7) A study of the standardization of diagnoses and diagnostic requirements in traditional Korean medicine III [13]; (8) The traditional Korean medical textbook on the digestive system [14]; (9) The traditional Korean medical textbook on the cardiovascular system [15]; and (10) Traditional Chinese medicine for encephalopathy [16]. Furthermore, we investigated clinical articles associated with stroke in the Korean literature, and then developed a set of necessary pattern indicators. Unnecessary pattern indicators included motor disability, dysphagia, dysarthria, and disturbances of consciousness. Although these indicators are primary symptoms of stroke, they were unhelpful in identifying a TKM pattern.

### Clinical application of pattern indicators

We clinically applied these pattern indicators, verifying their usefulness and compatibility. Because the indicators were composed of old language, not all indicators fit the patients' symptoms. A total of 147 stroke patients, whose demographic parameters are presented in Table 1, were enrolled from these Oriental medicine hospitals: 2 Wonkwang University Oriental Medicine hospitals (WKU OHs) and Dae Jeon University Oriental Medicine Hospital (DJU OH). All patients provided informed consent under procedures approved by the respective Institutional Review Boards (IRB NO: I-0910/02-004).

**Table 1:** Demographic parameters of study subjects

Characteristics	Male	Female
number	71	76

Age(years)	62.01 ± 11.96	66.72 ± 10.05

Inclusion criteria were as follows: stroke patients within 30 days of symptom onset, confirmed by imaging, such as computerized tomography (CT) or magnetic resonance imaging (MRI). Exclusion criteria were as follows: traumatic stroke patients, such as subarachnoid, subdural and epidural hemorrhaging patients. Two TKM doctors independently completed the case report form (CRF). The clinical trial period ranged from May 1, 2005 to May 30, 2005.^Q3^

### Expert committee approval

We organized a TKM expert committee (EC), which was launched on January 25, 2005, for our study: the fundamental study for the standardization and objectification of pattern identification in TKM for stroke (SOPI-Stroke). The EC was comprised of 19 members who majored in TKM from 11 Oriental medicine hospitals. More detailed description of the EC is presented in Table 2. At the first meeting of the EC that played a leading role in our study, the committee discussed the necessity of this project and the study design.

**Table 2:** The Member of Expert Committee

Name	Affiliation	Major, Position	Name	Affiliation	Major, Position
Byung Soon Moon	WKU	DCIM in TKM, professor	Ki Ho Cho	KHU	DCIM in TKM, professor

Seong Gyu Ko	KHU	DCIM in TKM, professor	In Lee	PNUKM	DCIM in TKM, professor

Jung Nam Kwon	DEU	DCIM in TKM, professor	Bum Sang Shim	KHU	DOPM in TKM, professor

Yun Sik Kim	DJU	DCIM in TKM, professor	Sang Kwan Lee	WKU	DCIM in TKM, professor

Sang Kwan Moon	KHU	DCIM in TKM, professor	Eun Chul Lim	DSOH	DSCM in TKM, head

Se Jin Park	DSOH	DCIM in TKM, head	In Soo Jang	WSU	DCIM in TKM, professor

Jong Hyung Park	KWU	DCIM in TKM, professor	Chan Yong Jun	KWU	DCIM in TKM, professor

Chi Sang Park	DGU-2	DCIM in TKM, professor	Chang Ho Han	DGU-1	DCIM in TKM, professor

In Chan Seol	DJU	DCIM in TKM, professor	Seok Hong	DSU	DCIM in TKM, professor

Gil Cho Shin	DGU-1	DCIM in TKM, professor			

KHU; Kyung Hee University, DEU; Dong Eui University, DJU; Dae Jeon University, DSOH; Dong Seo Oriental Hospital, KWU; Kyung Won University, DGU-1; Dong Guk University, DGU-2; Dae Gu University, PNUKM; Pusan National University, school of Korean Medicine, WKU; Wonkwang University, WSU; Woo Suk University, DSU; Dong Shin University

DCIM; Department of Circulatory Internal Medicine, DOPM; Department of Oriental Pathology Medicine, DSCM; Department of Sasang Constitutional Medicine,

### The case report form and standard operating procedures

We compiled the CRF to include the D-SPI and basic patient information. The CRF was composed of short-form questions in Korean for all clinicians to identify patterns without prejudice or difficulty. We developed standard operating procedures (SOPs) based on the CRF. All clinicians and researchers involved in this study were educated on the CRF and SOPs twice yearly to remove difficulties and misunderstandings and to enhance the concordance rate. We notified all clinicians of contested issues presented in the education process to minimize individual prejudices and enhance consistency.

## Results

### Definition of stroke in TKM and sets of subtypes

Researchers composed the stroke identification patterns and subtypes of pattern identification from the related literature, preliminary study and expert advice. The results were then agreed upon by the Korean Medical Stroke Diagnosis Standard Committee. Based on several theories for stroke identification in the TKM literature, all possible patterns for stroke were surveyed. Stroke was defined as focal neurological-deficit symptoms from cerebral circulatory disorders, including unconsciousness, hemiplegia, sluggish speech, numbness of the skin and other symptoms. Five patterns were identified: the Fire-heat pattern, the Dampness-phlegm pattern, the Yin deficiency pattern, the Qi deficiency pattern, and the Blood stasis pattern.

#### Fire-heat pattern

The Fire-heat pattern is characterized by any symptom of heat or fire that is contracted externally or engendered internally. This symptom can cause stroke through intense pathogenic heat and high fever. It is generally treated by externally clearing heat or internally eliminating fire.

#### Dampness-phlegm pattern

The Dampness-phlegm pattern is characterized by impeding Qi movement and its turbidity, heaviness, stickiness and downward-flowing properties. This symptom is due to the accumulation of damp phlegm in the lung and spleen in TKM. This pattern is caused by stroke and circulatory disturbances.

#### Blood stasis pattern

The Blood stasis pattern is characterized by blood stagnation, including extravagated blood and sluggish blood circulation or viscous or congested blood, all of which may become pathogenic factors.

#### Qi deficiency pattern

The Qi deficiency pattern is characterized by qi deficiency with diminished internal organ function, which is marked by shortness of breath, lassitude, listlessness, spontaneous sweating, a pale tongue and a weak pulse.

#### Yin deficiency pattern

The Yin deficiency pattern is characterized by yin deficiency with diminished moistening and the inability to restrain yang, which is usually manifested as fever.

### Draft construction by the initial adjustment of selected indicators

Overall, pattern indicators were obtained from the above procedures (Table 3). The pattern indicators were reorganized into 22 items. These items allowed TKM doctors to easily perform four examinations in stroke patients. The 22 items were: physique, headache, dizziness, complexion, eyes, tinnitus, mouth, tongue diagnosis, throat, sputum, chest, palpitation, abdomen, skin, extremities, pulse diagnosis, digestion, defecation, urine, insomnia, temperature and sweating. These items were systematically organized from the head to the foot according to the table of contents in the Dongeuibogam, the well-known encyclopedia of TKM [17]. Dongeuibogam (Treasured Paragon of Eastern Medicine) After the Japanese Invasion in year 1592, Heo, Joon received orders from King Seonjo to consolidate data with Jung, Jak; Yang, Ye-su; Kim, Eung-tak; Lee, Myeong-won; and Jung, Ye-nam to write Dongeuibogam. Dongeuibogam is very important book in TKM several aspects. After all procedures were completed, a draft document was composed for the standardization of pattern identification for stroke (D-SPI). Overall, 122 indicators of pattern identification in stroke were obtained, excluding common symptoms, such as exercise, consciousness, and language disorders. After the selection, the indicators were integrated with the indicator for every cause and translated into Korean. When indicators had uncertain meanings, they were written in Chinese characters.

**Table 3:** Pattern indicators

Number	Item		Pattern Indicator	Freq. (%)
**1**	body type		underweight	35 (23.8)
		
			overweight	36 (24.48)

**2**	headache	time	severe and sudden headache	5 (3.4)
		
			old headache	14 (9.52)
		
			continuous pain	20 (13.6)
		
		site	headache in the whole head	22 (14.96)
		
			site-fixed headache	8 (5.44)
		
			radiating headache	3 (2.04)
		
		aspect	severe and seems to be bursting	4 (2.72)
		
			tightened feeling	8 (5.44)
		
			headache with a pulling sensation	5 (3.4)
		
			deterioration of headache by fatigue	5 (3.4)
		
			hot head	3 (2.04)
		
			headache with anger	5 (3.4)
		
			heavy-headedness	25 (17)
		
			an unpleasant sensation with an urge to vomit and pain in the head	6 (4.08)
		
			un-refreshed head	29 (19.72)
		
			headache accompanied by stabbing pain	5 (3.4)
		
			headache accompanied by empty pain	6 (4.08)
		
			headache accompanied by a hot flush	3 (2.04)

**3**	dizziness		severe and accompanied nausea and vomiting	6 (4.08)
		
			slight dizziness	41 (27.89)

**4**	facial complexion		reddened complexion	37 (25.17)
		
			dark face discoloration	36 (24.48)
		
			black face with black eyelids	21 (14.28)
		
			white complexion	23 (15.64)
		
			pale face and red zygomatic site	20 (13.6)

**5**	eye's abnormal condition		red eyes	31 (21.08)
		
			purpura in the sclera	4 (2.72)
		
			dry eyes	11 (7.48)
		
			excessive gum in the corner of the eye	11 (7.48)

**6**	tinnitus		tinnitus is incidental	1 (0.68)
		
			tinnitus is continuous	4 (2.72)
		
			tinnitus is intermittent	7 (4.76)

**7**	tongue and mouth		bitter taste in the mouth	45 (30.61)
		
			thirst in the mouth	48 (32.65)
		
			aphta and tongues sores	4 (2.72)
		
			excessive saliva in the mouth	17 (11.56)
		
			cyanotic lips	17 (11.56)
		
			dry mouth	77 (52.38)
		
			fetid mouth odor	26 (17.68)

**8**	sputum		sticky sputum	18 (12.24)
		
			phlegm rale	32 (21.76)

**9**	oppression in the chest		heat vexation in the chest	10 (6.8)
		
			feeling of oppression in the chest	21 (14.28)
		
			bloated feeling in the chest and hypochondriac region	1 (0.68)

**10**	palpitations and fearful throbbing		palpitations and shortness of breath	(0)
		
			palpitation with anxiety or discomfort	(0)

**11**	abdomen		tenderness and fever in abdominal diagnosis	6 (4.08)
		
			sounds heard in abdominal diagnosis	17 (11.56)
		
			tenderness of the lower abdomen and accompanied pain	22 (14.96)
		
			mass in the abdomen	6 (4.08)
		
			no resistance to touch of the abdomen	44 (29.93)
		
			tension is felt when pressing the abdomen	40 (27.21)

**12**	skin		burning skin sensation	7 (4.76)
		
			attachment sensation of derma	18 (12.24)
		
			purpura	7 (4.76)
		
			feeling like insects' crawling	7 (4.76)
		
			feeling chilly on the skin	16 (10.88)
		
			dry skin	31 (21.08)

**13**	palm and sole		vexing heat in the extremities	14 (9.52)
		
			a subjective heaviness sensation of the body	76 (51.7)
		
			a pronounced cold in the extremities up to the knees and elbows or beyond	26 (17.68)
		
			a lack of physical strength in the four extremities	75 (51.02)
		
			heat in the palms and soles	20 (13.6)

**14**	digestion		stomach feels full	19 (12.92)
		
			feeling gastric reflux	9 (6.12)
		
			excessive appetite with increased food intake and recurrence of hunger sensation shortly after eating	4 (2.72)
		
			an unpleasant sensation with an urge to vomit	17 (11.56)
		
			loss of appetite	41 (27.89)

**15**	feces		hardened feces difficult to evacuate	36 (24.48)
		
			discharge of soft, unformed stools: a loose stool	7 (4.76)

**16**	urine		dark yellow or reddish urine	49 (33.33)
		
			a high volume of transparent urine	(0)
		
			failure of voluntary control of urination	49 (33.33)

**17**	sleeping		inability to sleep well due to fever and oppression	10 (6.8)
		
			excessive sleepiness night and day	4 (2.72)
		
			inability to sleep or abnormal wakefulness: insomnia	7 (4.76)
		
			inability to sleep due to anxiety	9 (6.12)

**18**	heat condition		high fever	34 (23.12)
		
			tidal fever	4 (2.72)

**19**	sweat		profuse sweating with fever: sweating when having fever	38 (25.85)
		
			lack of physical strength, excessive sweating during the daytime with no apparent cause, such as physical exertion, hot weather, thick clothing or medication	24 (16.32)
		
			night sweating	21 (14.28)

**20**	vocal sound energy		inclined to speak or speaking at a high volume	58 (39.45)
		
			disinclined to speak or speaking at a low volume	37 (25.17)

**21**	tongue		a larger than normal tongue, pale in color and delicate, usually bearing dental indentations on the margin-enlarged tongue	9 (6.12)
		
			a tongue with dental indentations on its margin: a teeth-marked tongue	40 (27.21)
		
			a tongue thinner than normal	11 (7.48)
		
			a tongue redder than normal, indicating the presence of heat	56 (38.09)
		
			a tongue with thorn-like protrusions on the surface	10 (6.8)
		
			a cyanotic tongue, indicating blood stasis or heat toxin in the nutrient-blood	17 (11.56)
		
			a tongue with red, white or black spots as well as thorn-like protrusions on its surface	9 (6.12)
		
			a tongue less red than normal, indicating qi and blood deficiency or the presence of cold deficiency	74 (50.34)
		
			dry tongue	25 (17)
		
			mirror tongue	17 (11.56)
		
			thin fur	76 (51.7)
		
			thick fur	26 (17.68)
		
			tongue coating white in color	67 (45.57)
		
			tongue coating yellow in color	41 (27.89)
		
			tongue coating black in color	1 (0.68)

**22**	pulse		floating pulse	26 (17.68)
		
			sunken pulse	33 (22.44)
		
			slow pulse	15 (10.2)
		
			rapid pulse	13 (8.84)
		
			strong purse	26 (17.68)
		
			weak purse	28 (19.04)
		
			string-like pulse	27 (18.36)
		
			slippery pulse	33 (22.44)
		
			fine pulse	20 (13.6)
		
			rough pulse	2 (1.36)

### Korea standard pattern identification for stroke I

**Figure 1:**
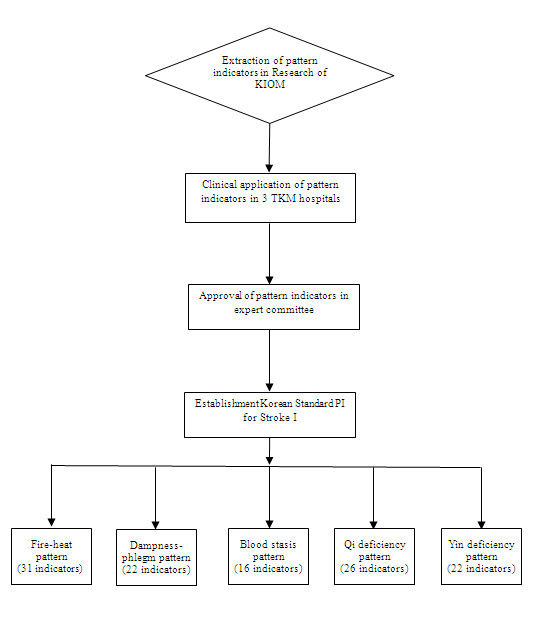
**Flow chart of our research**.

Based on the originally developed indicators, symptoms written in the foreign literature were found to have the same meaning as in the Korean literature. To determine frequency, we implemented a month-long preliminary study on 147 patients (June, 2005) in three Oriental medicine hospitals (Table 3). After this clinical field test, some pattern indicators were separated or combined, which led. Few indicators were eliminated. The remaining indicators were then discussed. The researchers developed consensus upon the Korea standard pattern identification for stroke I (K-SPI-Stroke I) to be presented at the Korean Medical Stroke Diagnosis Standard Committee on July 2005 (Figure 1), (Tables 4, 5, 6, 7 and 8).

**Table 4:** Indicators of the Fire-heat pattern (31)

Item	pattern indicator
headache	radiating headache(both temporal region and parietal region)
	
	severe and seems to be bursting
	
	hot head
	
	headache with anger
	
	severe and sudden headache
	
	seems to break and to be bursting

dizziness	severe and accompanied nausea and vomiting

facial complexion	reddened complexion

eye's abnormal condition	red eyes

tinnitus	ringing in the ear strongly

tongue and mouth	bitter taste and thirst in the mouth
	
	aphta and tongue sores
	
	fetid mouth odor

sputum	sticky and yellow sputum

oppression in the chest	heat vexation in the chest

palpitation and fearful throbbing	palpitation quickly
	
	palpitation with oppression

abdomen	tenderness and fever in abdominal diagnosis

skin	burning skin sensation

palm and sole	vexing heat in the extremities

digestion	feeling full and feeling gastric reflux in stomach
	
	excessive appetite with increased food intake and recurrence of hunger sensation shortly after eating

feces	hardened feces difficult to evacuate

urine	reddish urine and does not come out easily

sleeping	inability to sleep well due to fever and oppression

sweating	profuse sweating with fever: sweating when having fever

tongue	red tongue
	
	a tongue with thorn-like protrusions on its surface
	
	thick and yellow fur
	
	dry and black fur

purse	surging and rapid purse

**Table 5:** Indicators of the Dampness-phlegm pattern (22)

Item	pattern indicator
headache	heavy -headedness
	
	an unpleasant sensation with an urge to vomit and pain in the head
	
	un-refreshed head
	
	tightened feeling
	
	headache in whole head

dizziness	severe and accompanied nausea and vomiting

facial complexion	dark face discoloration
	
	black face with black eyelids

tinnitus	ringing in the ear strongly

mouth and lip	excessive saliva in the mouth

sputum	phlegm rale
	
	sticky and yellow sputum

oppression in the chest	feeling of oppression in the chest

palpitation and fearful throbbing	palpitation with oppression

abdomen	sounds heard in abdominal diagnosis

skin	attachment sensation of derma

digestion	an unpleasant sensation with an urge to vomit

tongue	larger than normal tongue, pale in color and delicate, usually bearing dental indentations on the margin-enlarged tongue
	
	a tongue with dental indentations on its margin: a-teeth -marked tongue
	
	thick and yellow or white fur

purse	surging purse

**Table 6:** Indicators of the Blood stasis pattern (16)

Item	pattern indicator
headache	headache with a pulling sensation
	
	site-fixed headache
	
	old headache

facial complexion	black face with black eyelid

eye's abnormal condition	purpura in the sclera

mouth	cyanotic lips

sputum	fishy smell mouth odor

oppression in the chest	bloated feeling in the chest and hypochondriac region

palpitations fearful throbbing	palpitation with anxiety or discomfort

abdomen	tension in the upper abdomen and complain of lower abdominal tenderness
	
	mass in the abdomen

skin	purpura

tongue	tongue purple in color
	
	tongue with red, white or black spots as well as thorn-like protrusions on its surface
	
	cyanotic tongue

Pulse	rough pulse

**Table 7:** Indicators of the Qi deficiency pattern (26)

Item	pattern indicator
headache	continuous pain
	
	headache accompanied by empty pain
	
	deterioration of headache by fatigue

dizziness	slight dizziness

facial complexion	white complexion

tinnitus	ringing in the ear slightly

sputum	spitting phlegm with a low viscosity

palpitations fearful throbbing	palpitations and shortness of breath

abdomen	no resistance to touch of the abdomen

skin	feeling like insects' crawling
	
	feeling chilly on the skin

palm and sole	a pronounced cold in the extremities up to the knees and elbows or beyond, also the same as cold extremities
	
	a lack of physical strength in the four extremities

digestion	loss of appetite

feces	discharge of soft, unformed stools: loose stool

urine	a high volume of transparent urine
	
	failure of voluntary control of urination

sweat	lack of physical strength, excessive sweating during the daytime with no apparent cause such as physical exertion, hot weather, thick clothing or medication

voice sound	disinclined to speak at a low volume

tongue	larger than normal tongue, pale in color and delicate, usually bearing dental indentations on the margin: enlarged tongue
	
	tongue with dental indentations on its margin: a-teeth-marked tongue
	
	a tongue less red than normal
	
	thin fur

pulse	a pulse that is deep, soft, thin and forceless

**Table 8:** Indicators of the Yin deficiency pattern (22)

Item	pattern indicator
headache	headache accompanied by hot flush
	
	continuous pain
	
	headache accompanied by empty pain

dizziness	dizziness slightly

facial complexion	pale face and red zygomatic site

tinnitus	ringing in the ear slightly

mouth and lip	dry mouth
	
	aphta and tongues sores

sputum	sputum with blood

palpitation and fearful throbbing	palpitation with anxiety or discomfort

abdomen	no resistance to touch of the abdomen

skin	dry skin

palm and sole	heat in the palms and soles

sleeping	inability to sleep due to anxiety

heat condition	afternoon tidal fever

sweat	night sweating

tongue	a tongue thinner than normal
	
	dry and red tongue
	
	thin fur or mirror tongue
	
	peeled fur

purse	fine and rapid tongue

The principles for developing indicators were as follows:

1) To reflect characteristic stroke symptoms

2) To clinically identify stroke patterns based on the present state of TKM

3) To consider associations with previous studies

4) To reflect the recent trend of stroke in the TKM literature

## Discussion

TKM occupies an independent position guaranteed by Korean medical laws [1]. Korea has is one national TKM school and 11 traditional Korean medicine colleges offering 6-year courses. Both the national school and colleges have their own hospitals, the majority of which are filled with a considerable number of stroke patients. Thus, we chose to standardize the TKM method of stroke diagnosis. One of the decisive procedures before stroke treatment is pattern identification, which determines the therapeutic method, such as acupuncture or herbs[18]. Pattern identification in stroke is a series of collection procedures that involves not only specific neurological deficits but also unspecific symptoms and indicators obtained by four examinations as well as determining treatment goals after integrating all data. When the cause and disease conditions are determined using pattern identification, TKM doctors adopt proper therapeutic methods to restore the imbalance [19]. Specific and unspecific symptoms do not tend to be visualized or digitized but assessed comparatively and synthetically during the diagnosis process [20]. Despite this feature, a standardized diagnosis is vital for TKM. Since 1996, China has endeavored to establish standardized diagnoses to establish new criteria [12]. However, neither developmental processes nor clinical verifications were found in the literature. This study involved verifying and standardizing clinical indicators and patterns from the classical literature. Several types of pattern identification exist, such as the cold-heat, deficiency-excess, visceral, and constitutional patterns of identification. Therefore, the first step towards standardizing pattern identification was to select only the patterns of identification most frequently observed. The diagnosis and treatment of stroke in Korea have been influenced by the publication of the Dongeuibogam, which contains medical theories of successive generations and clinical experiences from the 17th century [17]. Additionally, on halfway through the medical exchange between China and Korea, the blood stasis pattern concept was introduced. Currently, the wind, fire-heat, dampness-phlegm, blood stasis, and deficiency patterns are prevalent in clinics [4]. We constructed the patterns of Korean stroke considering pathological changes in TKM. Wind was excluded from the study because it is a pattern that explains the condition of a patient rather than the cause of stroke. Because deficiency has excessive sub-deficiency patterns, qi deficiency and yin deficiency, which were thought to be meaningful in investigating stroke, were included exclusively. The following step was to determine what clinical indicators belonged to each identification pattern and compose a draft from the clinical data. First, primary headings were sorted from the Dongeuibogam, and clinical indicators were supplemented from the ten academic sources frequently referred to by TKM doctors. A field test confirmed the frequency and difference between significant and insignificant indicators. Then, the Korea standard pattern identification for stroke I (K-SPI-Stroke I) was produced. Through the field test, we were able to investigate several indicators. Some indicators in the literature were unhelpful pattern identification for stroke (e.g., underweight or overweight), and some indicators required adjustment. For example, the tinnitus category needed to differentiate tinnitus intensity rather than tinnitus aspects. Also, some indicators of purse were combined due to their common combined use in clinical. It is notable that a consensus on clinical indicator measurement was obtained. Even doctors with the same medical education can potentially conduct different assessments. Therefore, we developed the CRF and SOP to standardize and digitize clinical indicators.

A limitation of our study was that the concepts of reliability and validity, which are used to assess diagnosis criteria in modern western medicine [21], were not introduced. Additionally, the concepts of patterns and clinical indicators are rooted in the TKM literature and can generate divergences among the literature from China, Japan, and other countries in eastern Asia. Some indicators were assessed considering the feature of Korean people.

## Conclusions

A strength of this study was that we determined 5 patterns and 117 clinical indicators composed of high-frequency indicators and stroke symptoms. We aimed to determine the significant clinical indicators and distinguishing patterns of stroke. Greater focus was placed on indicators of pathological conditions than on those of physiological conditions, and we endeavored to determine clinical significance by conducting a field test and discussions with experts. Given the high probability of different levels of experience among the experts, we first produced the CRF and SOP and placed importance on education and training to eliminate differences between experts. Consequently, we developed a systematic questionnaire after the literature search, expert consensus, and a field test, which provided an example for an objective and standardized pattern identification for stroke in TKM. We shall be able to develop various standardized differentiations of symptoms and indicators that fit the actual conditions of other disease.

## Competing interests

The authors declare that they have no competing interests.

## Authors' contributions

TYP conceived the study design. TYP, TWM surveyed and reviewed all pattern indicators. JAC, BKK, MMK searched and analyzed the literature. JAL drafted the manuscript. MSL and JSL helped with the previous study and critically reviewed the manuscript. All authors read and approved the final version of the manuscript.
